# *In Vitro* Cytotoxicity Induced by the Bufadienolides 1α,2α-Epoxyscillirosidine and Lanceotoxin B on Rat Myocardial and Mouse Neuroblastoma Cell Lines

**DOI:** 10.3390/toxins11010014

**Published:** 2019-01-02

**Authors:** Danielle Henn, Annette Venter, Christo Botha

**Affiliations:** Department of Paraclinical Sciences, Faculty of Veterinary Science, University of Pretoria, Onderstepoort 0110, South Africa; DanielleHenn@tuks.co.za (D.H.); annette.venter@up.ac.za (A.V.)

**Keywords:** cumulative bufadienolide, cytotoxicity, 1α,2α-epoxyscillirosidine, lanceotoxin B, neurotoxic, ultrastructure

## Abstract

Consumption of bufadienolide-containing plants are responsible for many livestock mortalities annually. Bufadienolides are divided into two groups; non-cumulative bufadienolides and cumulative bufadienolides. Cumulative bufadienolides are referred to as neurotoxic, as the chronic intoxication with this type of bufadienolide results in a paretic/paralytic syndrome known as ‘krimpsiekte’. The *in vitro* cytotoxicity of a non-cumulative bufadienolide, 1α,2α-epoxyscillirosidine, and a cumulative bufadienolide, lanceotoxin B, were compared using the MTT ((3-(4,5-dimethylthiazol-2-yl)-2,5-diphenyltetrazolium bromide reduction) assay after exposing rat myocardial (H9c2) and mouse neuroblastoma (Neuro-2a) cell lines. The effect of these two bufadienolides on cell ultrastructure was also investigated using transmission electron microscopy (TEM). H9c2 cells exhibited greater cytotoxicity when exposed to 1α,2α-epoxyscillirosidine, compared to lanceotoxin B. In contrast, Neuro-2a cells were more susceptible to lanceotoxin B. The EC_50_ (half maximal effective concentration) of lanceotoxin B exposure of Neuro-2a cells for 24–72 h ranged from 4.4–5.5 µM compared to EC_50_s of 35.7–37.6 µM for 1α,2α-epoxyscillirosidine exposure of Neuro-2a cells over the same period. 1α,2α-Epoxyscillirosidine induced extensive vacuolization in both cell types, with swollen RER (rough endoplasmic reticulum) and perinuclear spaces. Lanceotoxin B caused swelling of the mitochondria and sequestration of cytoplasmic material within autophagic vesicles. These results corroborate the notion that cumulative bufadienolides are neurotoxic.

## 1. Introduction

Cardiac glycosides are found in plants worldwide and based on their chemical structure are classified as either bufadienolides or cardenolides. Bufadienolide-containing plants are considered as one of the greatest causes of plant-associated poisoning of livestock in South Africa [1]. Bufadienolides are C-24 steroids with a double unsaturated six-membered lactone ring attached to C-17β. Bufadienolides can be further divided into non-cumulative and cumulative bufadienolides [2]. Depending on the type and dose of the bufadienolide-containing plant ingested by the animal, poisoning can manifest as either acute or chronic intoxication. 

Non-cumulative bufadienolides cause acute cardiac glycoside poisoning in animals, which affects the cardiovascular, gastrointestinal, respiratory and nervous systems. These non-cumulative bufadienolides can be found in plants belonging to the genera *Moraea* and *Drimia*, among others. *Moraea pallida* (yellow tulip) is well known for causing livestock poisoning and contains the non-cumulative bufadienolide 1α,2α-epoxyscillirosidine as its main toxic principle [3].

In contrast, cumulative bufadienolides can cause either acute or chronic intoxication depending on the dose and are present in the plants of the family Crassulaceae (‘plakkies’), specifically from the genera *Tylecodon*, *Cotyledon* and *Kalanchoe* [2]. Although bufadienolide-containing plants have a worldwide distribution, plants containing cumulative bufadienolides have, to date, only been reported in Southern Africa. During chronic intoxication the clinical signs associated with the cardiovascular, gastrointestinal and respiratory systems are less apparent, while the nervous signs predominate. The chronic form of intoxication is a paretic condition known colloquially as ‘krimpsiekte’ and occurs most often in small stock [1]. Affected animals assume a characteristic posture, from which the name ‘krimpsiekte’ is derived, by standing with their back arched, feet together and head bowed down. Lanceotoxin B is a cumulative bufadienolide, isolated from *Kalanchoe lanceolata*, reportedly causing krimpsiekte in sheep and has a subcutaneous LD_50_ (half maximal lethal dose) of 0.10 mg/kg in guinea-pigs [4]. 

All cardiac glycosides, including bufadienolides, act by inhibiting the Na^+^/K^+^-ATPase (sodium/potassium-adenosine triphosphatase) causing an increase in the intracellular sodium concentration of the cell and subsequently indirectly inhibiting the Na^+^/Ca^2+^-exchanger. The ability of the Na^+^/K^+^-ATPase to act as a scaffold protein that regulates downstream effectors, as well as the increase in intracellular calcium, allow the cardiac glycoside to trigger various cell signaling pathways [5]. These pathways then proceed to influence cellular functions such as proliferation, cell death mechanisms, protein synthesis and cellular metabolism [5], and even cellular attachment [6,7]. In addition, it was proposed that cumulative bufadienolides can bind to the nicotinic acetylcholine receptors of the neuromuscular junction and that this is what causes the neuromuscular dysfunction [8]. Based on the cholinergic activity, it was hypothesized that cumulative bufadienolides occupy the neuromuscular receptors, decreasing their functionality. With continuous discharge, the acetylcholine stores of the motor nerve terminal are eventually depleted, and the remaining receptors are occupied with acetylcholine, desensitizing the receptors to further stimulation; as a result, fatigue sets in. This leads to the myasthenia gravis-like weakness seen in affected animals. Chronic intoxication with the cumulative bufadienolide, cotyledoside, has been shown to cause lesions in the white matter of thalamic nuclei, explaining some of the motor function deficiencies seen in affected animals [9].

The objective of this study was to investigate the *in vitro* cytotoxicity induced by 1α,2α-epoxyscillirosidine and lanceotoxin B on rat myocardial (H9c2) and mouse neuroblastoma (Neuro-2a) cell lines, to lend credence to the premise that cumulative bufadienolides are neurotoxic. Additionally, the ultrastructural changes caused by the non-cumulative and cumulative bufadienolide respectively, was investigated using electron microscopy.

## 2. Results

### 2.1. The Cytotoxicity of 1α,2α-Epoxyscillirosidine and Lanceotoxin B on H9c2 and Neuro-2a Cell Lines

The viability of H9c2 and Neuro-2a cells after exposure to a serial dilution of 1α,2α-epoxyscillirosidine and lanceotoxin B for 24, 48 and 72 h were examined using the MTT ((3-(4,5-dimethylthiazol-2-yl)-2,5-diphenyltetrazolium bromide reduction) viability assay. 

**Figure 1:**
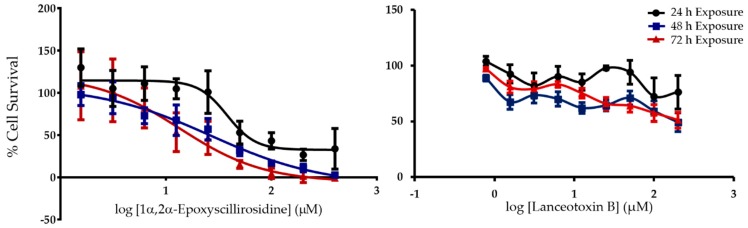
The semi-log concentration response curves of 1α,2α-epoxyscillirosidine and lanceotoxin B on H9c2 cells for 24, 48 and 72 h exposure expressed as percentage cell survival compared to the solvent control. Error bars indicate standard error of the mean.

When exposed to H9c2 cells, 1α,2α-epoxyscillirosidine induced a time-dependent effect, with decreasing EC_50_s after 24, 48 and 72 h exposure respectively (Table 1). The non-cumulative bufadienolide had a concentration-dependent effect as well with the percentage cell survival decreasing with higher toxin concentrations as depicted in the non-linear regression curves (Figure 1). A hormetic effect at low 1α,2α-epoxyscillirosidine concentrations was observed, with cell survival increasing to above that of the solvent control. In contrast to 1α,2α-epoxyscillirosidine, lanceotoxin B had a less potent effect on the H9c2 cells (Figure 1), with an EC_50_ greater than 200 µM at 24 h with the 95% CI (confidence interval) falling between 64% and 92% cell survival (Table 1). At 48 h, the EC_50_ was around 200 µM with a 95% CI of between 49% and 68% cell survival and finally, at 72 h the EC_50_ was below 200 µM with the 95% CI falling between 36% and 48% cell survival. A significant concentration-dependent effect could be seen at 48 and 72 h exposure, with the percentage cell survival decreasing with higher lanceotoxin B concentrations. At 24 h however, the concentration dependent effect was not significant.

**Figure 2:**
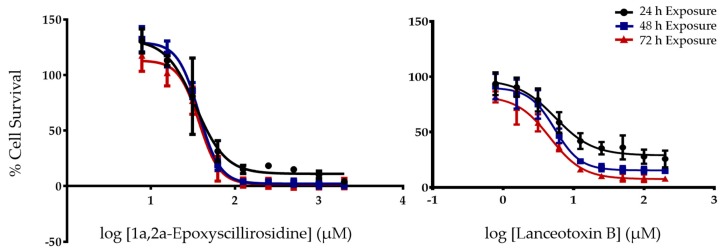
The semi-log concentration response curves of 1α,2α-epoxyscillirosidine and lanceotoxin B on Neuro-2a cells for 24, 48 and 72 h exposure expressed as percentage cell survival compared to the solvent control. Error bars indicate standard error of the mean.

When Neuro-2a cells were exposed to 1α,2α-epoxyscillirosidine, there were no significant time-dependent effects between the EC_50_s at 24, 48 and 72 h exposure. The EC_50_s of 1α,2α-epoxyscillirosidine did not significantly differ between exposure times (Table 1). The percentage cell survival decreased with higher 1α,2α-epoxyscillirosidine concentrations as shown with the non-linear regression curves (Figure 2). Lanceotoxin B had EC_50_s below 10 µM for all exposure times (Table 1). As with 1α,2α-epoxyscillirosidine, lanceotoxin B showed a clear concentration dependent effect, with higher concentrations reducing the Neuro-2a cell survival (Figure 2).

### 2.2. The Effect of 1α,2α-Epoxyscillirosidine and Lanceotoxin B on H9c2 and Neuro-2a Cell Line Ultrastructure 

**Figure 3:**
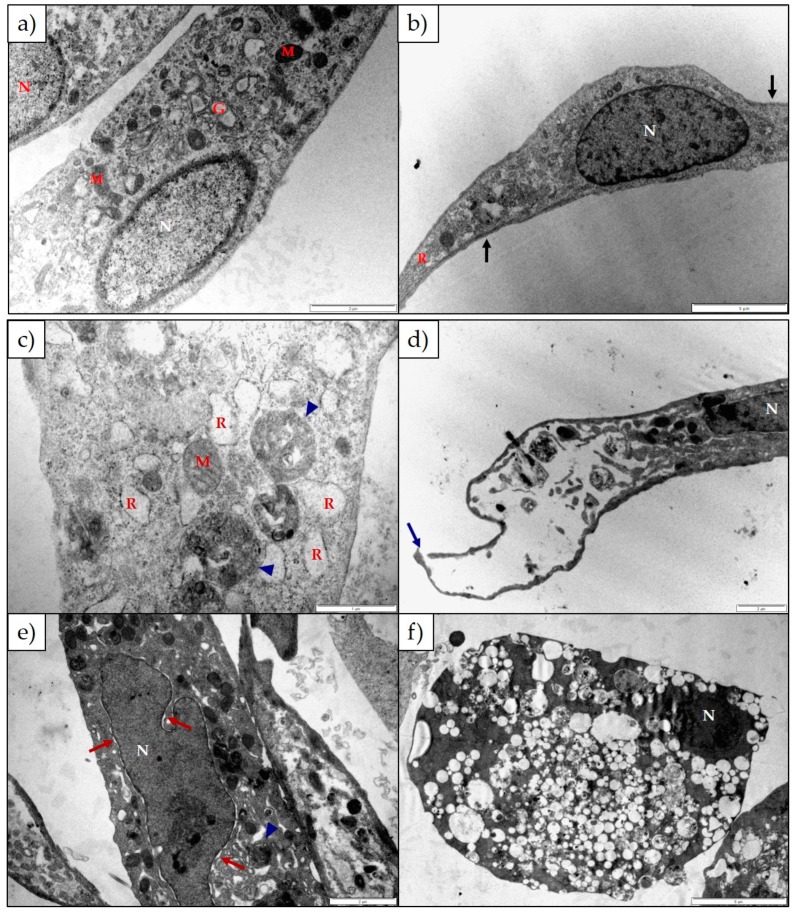
Electron micrograph of (**a**,**b**) untreated H9c2 cells and H9c2 cells exposed to (**c**) 25 µM and (**d**) 100 µM 1α,2α-epoxyscillirosidine for 24 h; (**e**) 100 µM 1α,2α-epoxyscillirosidine for 48 h; (**f**) 100 µM 1α,2α-epoxyscillirosidine for 72 h. The untreated cells were long and thin with tapered ends. The cytoskeleton was associated with the plasma membrane (black arrows). The H9c2 cells treated with 1α,2α-epoxyscillirosidine has plasma membrane damage (blue arrow), swollen RER and perinuclear spaces (red arrows) and cells were extensively vacuolated. Autophagic vesicles could be seen within the cytoplasm (blue arrow heads). G—Golgi Complexes; M—Mitochondria; N—Nuclei; R—Rough Endoplasmic Reticulum. The scale bar at the bottom right corner represents 5 µm (**b**,**f**), 2 µm (**a**,**d**,**e**) and 1 µm (**c**) respectively.

The ultrastructure of H9c2 and Neuro-2a cells after exposure to either 1α,2α-epoxyscillirosidine or lanceotoxin B for 24 h, 48 h and 72 h was investigated using TEM. Untreated H9c2 cells were long, thin and tapered at both ends (Figure 3a,b). The cytoskeleton was associated with the plasma membrane (black arrows), and nuclei were round or oval shaped. The RER, Golgi complexes and mitochondria were normal. After exposure to 1α,2α-epoxyscillirosidine the Golgi complexes and RER became swollen, with ribosomes dissociating from the RER (Figure 3c). The plasma membrane of the cells was damaged (Figure 3d), with some cellular debris visible. The nuclei were affected with swollen perinuclear spaces (red arrows; Figure 3e) and the nuclear material condensing at 48 and 72 h exposure. Autophagic vesicles (blue arrow heads; Figure 3c,e) could be seen distributed throughout the cell and the cytoplasm of the cells was extensively vacuolated (Figure 3f).

**Figure 4:**
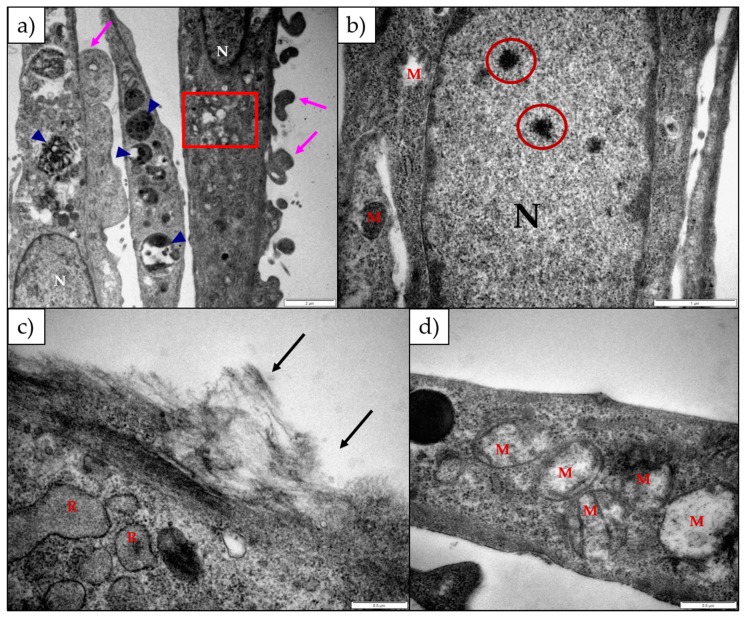
Electron micrograph of H9c2 cells exposed to (**a**) 25 µM and (**b**) 100 µM lanceotoxin B for 24 h; (**c**) 5 µM lanceotoxin B for 48 h; (**d**) 100 µM lanceotoxin B for 72 h. The cytoplasm contained autophagic vesicles (blue arrow heads), swollen Golgi complexes (red square) and swollen mitochondria (**d**). The nuclei were normal, except for some aggregates in the nucleoplasm (red circles). The cells formed plasma membrane blebs (pink arrows) and the cytoskeleton associated with the plasma membrane was disrupted (black arrows). M—Mitochondria; N—Nuclei; R—Rough Endoplasmic Reticulum. The scale bar at the bottom right corner represents 2 µm (**a**), 1 µm (**b**) and 0.5 µm (**c**,**d**) respectively.

The H9c2 cells exposed to lanceotoxin B had slightly swollen Golgi complexes (red square), while the RER remained unaffected (Figure 4a). The number of autophagic vesicles in the cytoplasm increased (blue arrow heads; Figure 4a). Many cells were shrunken, rounded and formed plasma membrane blebs indicative of apoptosis (pink arrows; Figure 4a). The mitochondria were damaged and became grossly swollen after longer exposure periods (Figure 4d). The nuclei appeared unaffected, but with some having electron-dense aggregates in the nucleoplasm (red circles; Figure 4b). The plasma membrane and associated cytoskeleton was disrupted (black arrows; Figure 4c). 

**Figure 5:**
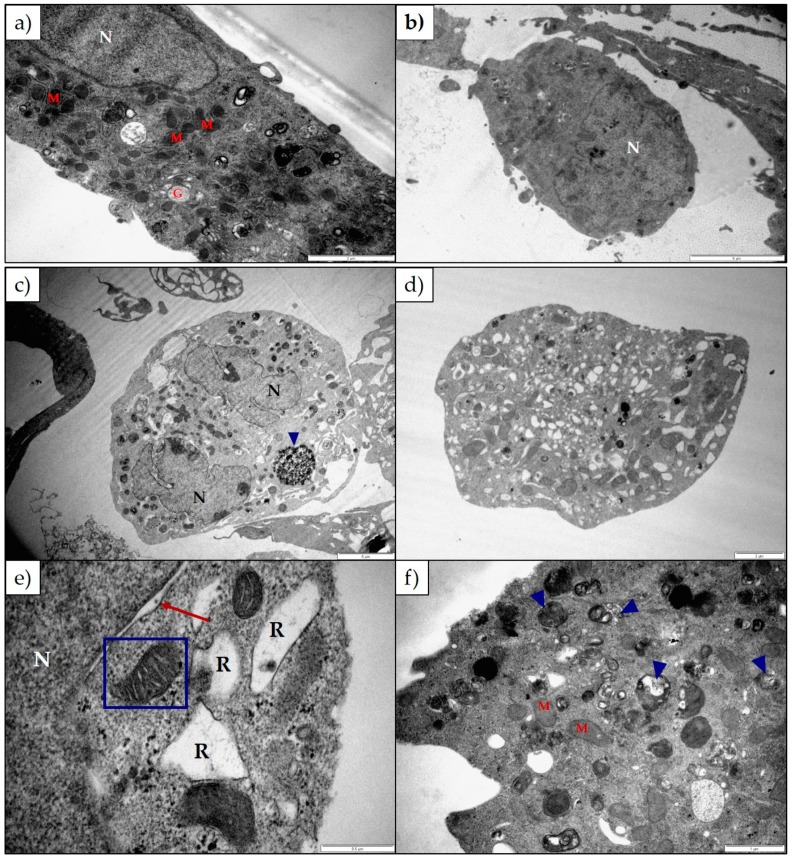
Electron micrograph of (**a**,**b**) untreated Neuro-2a cells and Neuro-2a cells exposed to (**c**) 100 µM 1α,2α-epoxyscillirosidine for 24 h; (**d**,**e**) 100 µM 1α,2α-epoxyscillirosidine for 48 h; (**f**) 100 µM 1α,2α-epoxyscillirosidine for 72 h. The organelles of untreated Neuro-2a cells appeared normal, with round or slightly convoluted nuclei. Cells exposed to 1α,2α-epoxyscillirosidine were extensively vacuolated, with swollen RER and perinuclear spaces. The cristae of some mitochondria were ballooned, and the nuclei were radially segmented. Many autophagic vesicles could be seen within the cytoplasm (blue arrow heads). G—Golgi complexes; M—Mitochondria; N—Nuclei; R—Rough Endoplasmic Reticulum. The scale bar at the bottom right corner represents 5 µm (**b**,**c**), 2 µm (**a**,**d**), 1 µm (**f**) and 0.5 µm (**e**) respectively.

Untreated Neuro-2a cells had clear Golgi complexes, mitochondria surrounded by RER and few autophagic vesicles within the cytoplasm (Figure 5a,b). Nuclei were round, with a few having a more convoluted shape. In contrast, Neuro-2a cells exposed to 1α,2α-epoxyscillirosidine for 24, 48 and 72 h indicated slightly swollen Golgi complexes, with swollen RER. Ribosomes dissociated from the RER and aggregated in the cytoplasm (Figure 5c). The nuclei of Neuro-2a cells exposed to 100 µM of 1α,2α-epoxyscillirosidine were radially segmented or irregularly shaped (Figure 5a). The cytoplasm was extensively vacuolated (Figure 5c,d), with autophagic vesicles (blue arrow heads; Figure 5c,f) being distributed throughout the cytoplasm. The mitochondria were damaged, while some had ballooned cristae (blue box; Figure 5e). As with the H9c2 cells, the nuclei of Neuro-2a cells exposed to 1α,2α-epoxyscillirosidine had swollen perinuclear spaces (red arrows; Figure 5e). 

**Figure 6:**
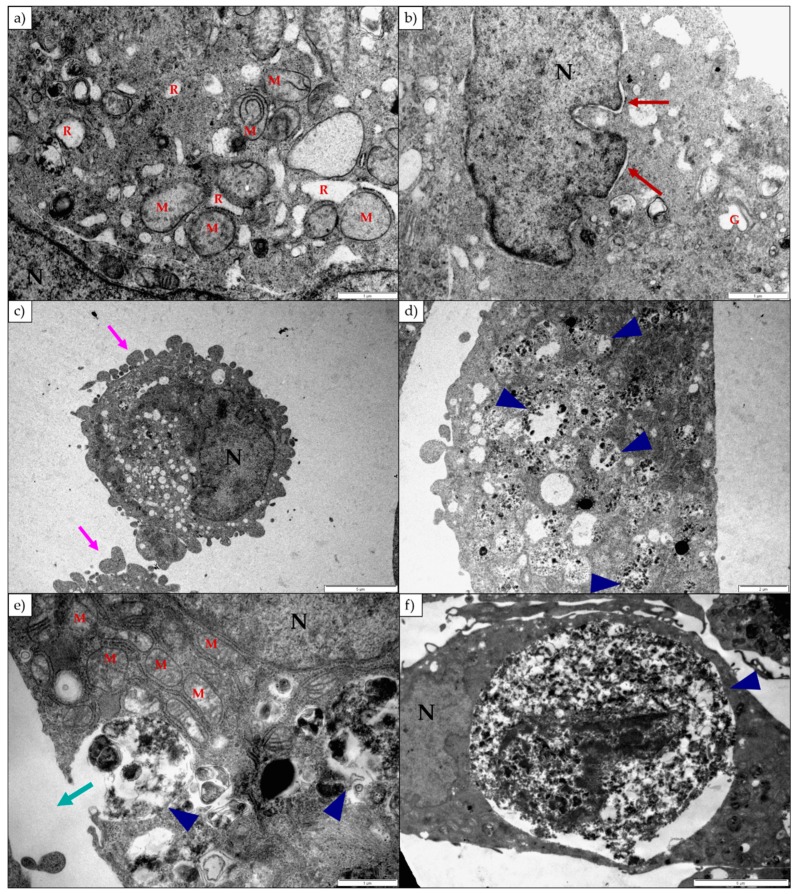
Electron micrograph of Neuro-2a cells exposed to (**a**,**b**) 5 µM and (**c**,**d**) 25 µM lanceotoxin B for 24 h; (**e**) 100 µM lanceotoxin B for 48 h; (**f**) 25 µM lanceotoxin B for 72 h. Cells had membrane blebs (pink arrows). At 5 µM after 24 h exposure cells showed swollen RER and perinuclear spaces. The mitochondria were grossly swollen (**a**,**e**). Cell content was sequestered in autophagic vesicles (blue arrow heads) and appeared to be released to the outside of the cells (cyan arrow). G—Golgi Complexes; M—Mitochondria; N—Nuclei; R—Rough Endoplasmic Reticulum. The scale bar at the bottom right corner represents 5 µm (**c**,**f**), 2 µm (**d**) and 1 µm (**a**,**b**,**e**) respectively.

In general, lanceotoxin B caused the Golgi complexes and mitochondria of the Neuro-2a cells to swell (Figure 6a,b,e). The RER remained unaffected, except at 24 h in Neuro-2a cells exposed to 5 µM lanceotoxin B (Figure 6a). Similarly, lanceotoxin B caused the perinuclear space of the nuclei to become swollen (red arrows; Figure 6b) at that concentration for 24 h exposure. The nuclei remained unaffected otherwise. Many cells were shrunken and rounded, forming membrane blebs (pink arrows; Figure 6c) and some showing plasma membrane damage. The number of autophagic vesicles (blue arrow heads; Figure 6d–f) in the affected cells increased in number. Large portions of the cell cytoplasm were sequestered in autophagic vesicles. Additionally, some of the autophagic vesicles extruded their content to the outside of the cell (cyan arrow; Figure 6e). 

## 3. Discussion

Due to the paretic/paralytic nature of chronic intoxication in small stock, the cumulative bufadienolides are often referred to as neurotoxic. We thus compared the *in vitro* cytotoxicity of a cumulative bufadienolide, i.e., lanceotoxin B with a non-cumulative bufadienolide, 1α,2α-epoxyscillirosidine on both myocardial (H9c2) and neuroblastoma (Neuro-2a) cell lines (Table 1). The H9c2 cells were more susceptible to 1α,2α-epoxyscillirosidine with the EC_50_ of lanceotoxin B being more than five times greater than that of 1α,2α-epoxyscillirosidine after 24 h exposure. In contrast, lanceotoxin B had EC_50_s below 6 µM at all three exposure times when Neuro-2a cells were assessed, also being much lower than the EC_50_s of 1α,2α-epoxyscillirosidine, ranging from 35.7 to 37.6 µM. These results support the theory that lanceotoxin B is neurotoxic, as lanceotoxin B induced greater cytotoxicity towards nerve cells. The neurotoxicity of the cumulative bufadienolides are believed to be due to their stereochemistry that differs from that of non-cumulative bufadienolides and cardenolides [10]. Unlike the other cardiac glycosides, cumulative bufadienolides have a levorotatory sugar strongly attached to the C3 position of the aglycone that cannot be removed by acid hydrolysis [10]. 

Of note, low concentrations of 1α,2α-epoxyscillirosidine had a positive effect on cell survival of both the H9c2 and Neuro-2a cell lines, showing a possible hormetic effect. Similar results have previously been reported when using low concentrations of the cardenolide, ouabain [11,12]. The toxicity of the bufadienolides falling within the micromolar range was not unexpected as rodent cells are less susceptible to cardiac glycosides compared to other animals [13,14]. 

The ultrastructural changes observed in both H9c2 and Neuro-2a cells exposed to 1α,2α-epoxyscillirosidine included swollen Golgi complexes, RER and enlarged perinuclear space (Figure 3 and Figure 5). These swollen organelles can be ascribed to the disruption of the ionic homeostasis of the cells caused by the bufadienolide. The RER and perinuclear space act as a storage site for intracellular calcium. The increased concentration of intracellular Na^+^ and Ca^2+^ ions are accompanied by the influx of water, causing the organelles to swell. In addition to mitochondrial damage, 1α,2α-epoxyscillirosidine caused some of the mitochondrial cristae to become slightly ballooned (Figure 5e), while the mitochondrial matrix remained unchanged. The inner and outer mitochondrial membranes have different permeability capacities thus causing the cristae and matrix to swell separately or subsequently. Exposure to 1α,2α-epoxyscillirosidine also caused large scale vacuolation of the cytoplasm (Figure 3 and Figure 5), also possibly due to the disruption of the ion homeostasis of the cell. These vacuoles seem to originate from the Golgi complexes and the RER. The swollen RER, in addition to the dissociated ribosomes clumping within the cytoplasm, suggested that protein synthesis was disrupted. 

Lanceotoxin B caused the Golgi complexes of both H9c2 and Neuro-2a cells to swell, also conceivably due to the disruption of the sodium, potassium and calcium homeostasis of the cells. However, in contrast to cells exposed to 1α,2α-epoxyscillirosidine, the RER of the H9c2 cells remained unaffected. The RER and nuclei of the Neuro-2a cells also remained mostly unaffected. The mitochondrial matrix of both H9c2 and Neuro-2a cells became grossly swollen after exposure to lanceotoxin B (Figure 4 and Figure 6). The swelling, as mentioned above, is due to the influx of water that accompanies the increased ion concentrations. The cytoskeleton associated with the plasma membrane of the H9c2 cells were also affected. Calcium activates different proteases, and these can subsequently degrade the cytoskeleton [15]. Lanceotoxin B caused a noticeable increase in the number of autophagic vesicles within the cytoplasm of affected cells. This was especially apparent in Neuro-2a cells, with large parts of the cells sequestered (Figure 6). Autophagy has historically been classified as a type of cell death, however its role in cell death is often misinterpreted [16]. Cells induce autophagy as a survival mechanism and often cell death is cell death with autophagy opposed to cell death by autophagy. Autophagy and neuronal cell death have previously been linked [17,18], thus corroborating the contention that lanceotoxin B is neurotoxic. The H9c2 cells exposed to lanceotoxin B were apoptotic, with shrunken, rounded cells; many with membrane blebs or what appears to be apoptotic bodies. In contrast, Neuro-2a cells exposed to lanceotoxin B, besides the extensive autophagy, also showed signs of both apoptosis and necrosis. The mixed morphological signs of cell death could be as a result of the various cellular pathways disrupted by the bufadienolides. 

In future, molecular studies can be performed to clarify the type of cell death caused by these cumulative bufadienolides which will contribute to our understanding of the mechanism of toxicity underlying ‘krimpsiekte’. In addition, investigating the ultrastructural changes in appropriate cells from poisoned animals and cells believed to not be affected by these bufadienolides could be of interest. 

## 4. Materials and Methods 

### 4.1. Cell Cultures

Rat myocardial (H9c2(2-1) (ATCC^®^ CRL-1446™)) and mouse neuroblastoma (Neuro-2a (ATCC^®^ CRL-131™)) cell lines were obtained from the ATCC (American Type Culture Collection). The cultures were grown in HyClone DMEM (Dulbecco’s Modified Eagle’s Medium)-High Glucose, supplemented with 4 mM glutamine, 1 mM sodium pyruvate, 10% fetal calf serum (Gibco, Life technologies, Carlsbad, CA, USA), 100 U/mL penicillin and 100 U/mL streptomycin (Lonza, Verviers, Belgium). The cells were maintained in an incubator at 37 °C and a humidified atmosphere of 5% CO_2._

### 4.2. Toxins

1α,2α-Epoxyscillirosidine and lanceotoxin B (>95% purity) used in this study were previously isolated from *M. pallida* and *K. lanceolata* respectively and were preserved in dried form and stored in a secure place as part of the natural toxin collection of the Department of Paraclinical Sciences, Faculty of Veterinary Science, Onderstepoort. The stock solutions of 1α,2α-epoxyscillirosidine were prepared in a 1:1 organic solvent-complete DMEM media mixture using acetone, while stock solutions for lanceotoxin B were prepared in DMSO (dimethyl sulfoxide).

### 4.3. Cell Survival Assays

The percentage cell survival of H9c2 and Neuro-2a cells after exposure to either 1α,2α-epoxyscillirosidine or lanceotoxin B was determined using the MTT assay [19]. The cells were seeded into a 96 well plates at a concentration of 5000 cells/well for the Neuro-2a cells and 10,000 cells/well for the H9c2 cells, respectively in 200 µL complete media per well, 24 h prior to the commencement of the exposure study. The cells were exposed to a 2× serial dilution of 1α,2α-epoxyscillirosidine and lanceotoxin B for 24, 48 and 72 h. The percentage solvent was kept constant for all dilutions at 0.5% solvent/well. After termination of the exposure studies and following rinsing of the wells with 200 µL PBS (phosphate buffered saline), pH 7.4, 20 µL 0.005 g/mL Thiazolyl Blue Tetrazolium Bromide dissolved in PBS (Sigma-Aldrich, St. Louis, MO, USA) and 200 µL complete DMEM media were added to each well. The plates were then incubated in the dark at 37 °C for 2 h, the MTT medium replaced with 100 µL DMSO and shaken for 5 min in the dark to solubilize the MTT formazan crystals. The absorbance of the MTT product was measured at 570 nm and the background at 630 nm using a Synergy HT BioTek microplate reader (BioTek Instruments, Inc., Winooski, VT, USA).

### 4.4. Statistical Analysis

Data were analyzed using Microsoft Excel (Office 365, Microsoft, Redmond, WA, USA) and GraphPad Prism (Version 6.0, GraphPad Prism Software Inc., La Jolla, CA, USA). All repeats of the assays were grouped together, and the outliers removed. The data was tested for compliance with normality and homogeneous variance using D’Agostino and Pearson omnibus normality tests and Bartlett’s test, respectively. An ANOVA (factorial analysis of variance) was used to see if there were significant differences (*p* > 0.05) between the EC_50_s at the different exposure periods; and in the case that there were, Student’s t-test was used. The 95% confidence intervals were calculated by the following equation:

95% CI = Average cell survival at highest concentration of lanceotoxin B ± (1.96× Standard error of the mean).

### 4.5. Transmission Electron Microscopy

The ultrastructural changes induced by the toxins on H9c2 and Neuro-2a cells were investigated using TEM. H9c2 and Neuro-2a cells were exposed to 5, 25 and 100 µM 1α,2α-epoxyscillirosidine and lanceotoxin B for 24, 48 and 72 h. After termination of the exposure, the cells were fixed with 2.5% glutaraldehyde in 0.0075 M sodium phosphate buffer (pH 7.4) for 1 h. The cells were scraped off from the plate, centrifuged at 950 g and rinsed three times for 10 min with 0.075 M phosphate buffer. The cells were post-fixed with osmium tetroxide and rinsed with the phosphate buffer for 10 min. The cells were serially dehydrated in 30%, 50%, 70%, 90% and three times in 100% ethanol. The samples were then imbedded in TAAB 812 epoxy resin [20] and sectioned using an ultra-microtome (Leica EM UC7). Each section was contrasted with 2% aqueous uranyl acetate for 10 min and lead citrate for 2 min [21]. The sections were examined using a Philips CM10 Transmission Electron Microscope (Philips, Amsterdam, The Netherlands).

## Figures and Tables

**Table 1:** The EC_50_ of 1α,2α-epoxyscillirosidine and lanceotoxin B on H9c2 and Neuro-2a cells after 24, 48 and 72 h exposure.

Cell Lines	Bufadienolide	24 h	48 h	72 h
H9c2	1α,2α-Epoxyscillirosidine	41.39 ± 4.37 µM *n* = 3	25.42 ± 3.73 µM *n* = 3	12.65 ± 2.75 µM *n* = 3
	Lanceotoxin B	>200 µM (95% CI: 64–92) * n = 4	~200 µM (95% CI: 49–68) * n = 4	<200 µM (95% CI: 36–48) * n = 4
Neuro-2a	1α,2α-Epoxyscillirosidine	35.73 ± 10.59 µM *n* = 3	37.56 ± 3.18 µM *n* = 3	37.35 ± 2.30 µM *n* = 3
	Lanceotoxin B	5.46 ± 0.37 µM *n* = 4	5.27 ± 0.59 µM *n* = 4	4.43 ± 0.67 µM *n* = 4

EC_50_ (µM) ± Standard error of the mean for at least three biological repeats (*n*). * At 200 µM the percentage cell survival fell between that indicated in brackets with a 95% confidence interval.
